# Supplementary material for: Stain-free detection of embryo polarization using deep learning
Source: Sci Rep. 2022 Feb 14;12:2404. doi: 10.1038/s41598-022-05990-6 (PMC8844381; doi:10.1038/s41598-022-05990-6)
Supplement: Supplementary file 1 — Supplementary Information. [file 41598_2022_5990_MOESM1_ESM.docx]

**Supporting Information**

**Stain-free Detection of Embryo Polarization using Deep Learning**

Cheng Shen^1,+^, Adiyant Lamba^2,+^, Meng Zhu^2,3^, Ray Zhang^4^, Changhuei Yang^1,5,*^, and Magdalena Zernicka Goetz^2,5,*^

^1^ Department of Electrical Engineering, California Institute of Technology, Pasadena, CA, USA

^2^ Mammalian Embryo and Stem Cell Group, Department of Physiology, Development and Neuroscience, University of Cambridge, Downing Street, Cambridge, CB2 3EG, UK

^3^ Blavatnik Institute, Harvard Medical School, Department of Genetics, Boston, MA 02115, USA

^4^ Department of Pathology and Immunology, Washington University School of Medicine, St. Louis, MO, USA

^5^ Division of Biology and Biological Engineering, California Institute of Technology, Pasadena, CA, USA

^+^ These authors contributed equally

^*^ co-corresponding authors: chyang@caltech.edu, magdaz@caltech.edu

**Including:**

Figures S1 to S6

Table S1, S2


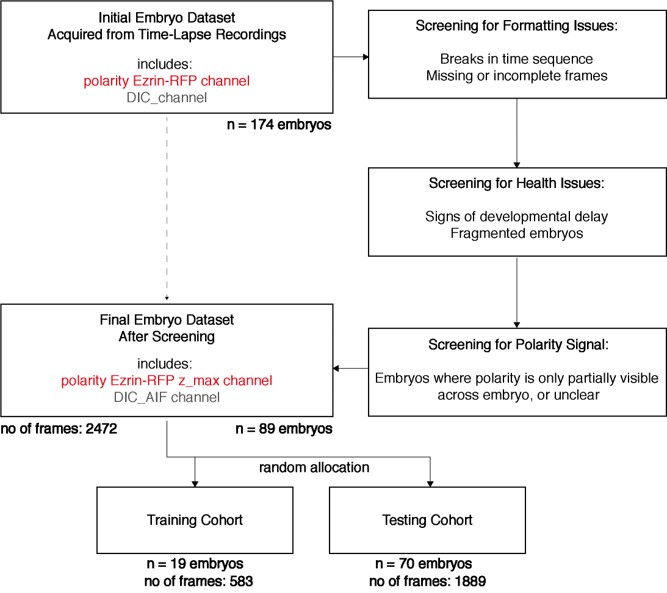


**Figure S1. Flowchart indicating initial cleaning and pre-processing of data.**

We analyzed 174 mouse embryo time-lapse recordings from dual-modal confocal microscope imaging, containing a DIC channel and a fluorescent polarity-indicating channel. After screening for image and embryo development quality, 89 embryos were left, with each channel compressed along the *z* axis using different algorithms and then randomly split into a training and testing cohort.


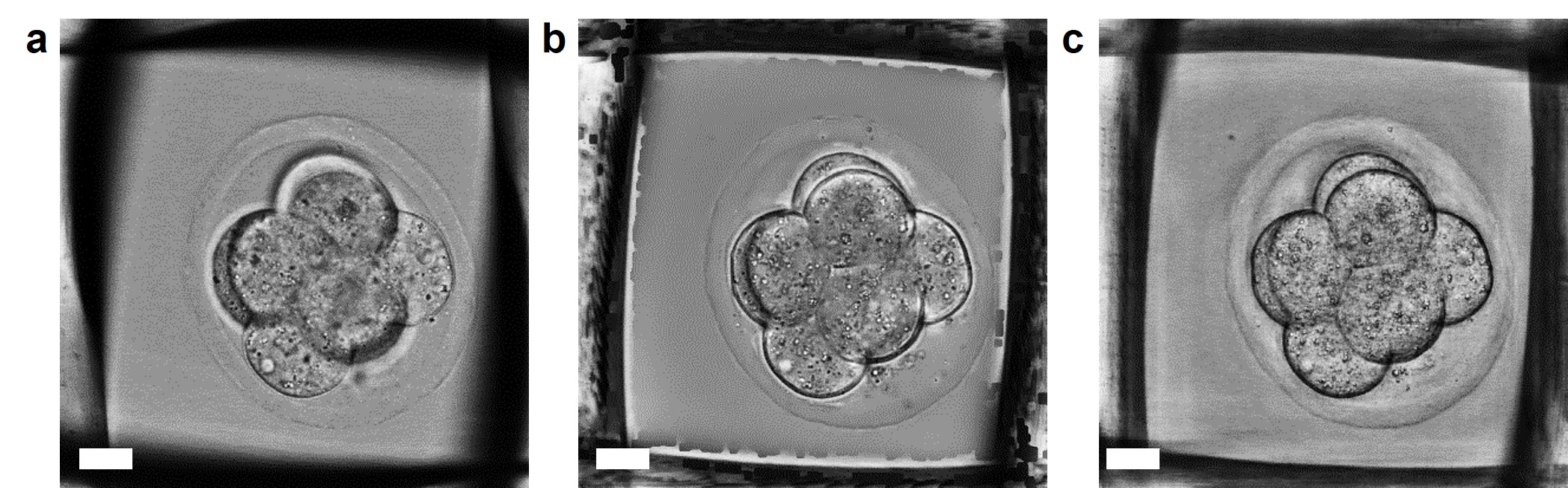


**Figure S2. Comparison among different input image formats.**

Previous deep learning studies on embryo development used a single *z* slice image, in most cases the middle plane - see (a). However, this resulted in some blastomeres being highly defocused and blurred. The traditional all-in-focus algorithm based on variance metric (b) can bring all the blastomeres into focus in a single 2D image but also result in some artifacts. Thus, we proposed to utilize the all-in-focus algorithm based on dual tree complex wavelet transform (c). Scale bar = 20 *μ*m.


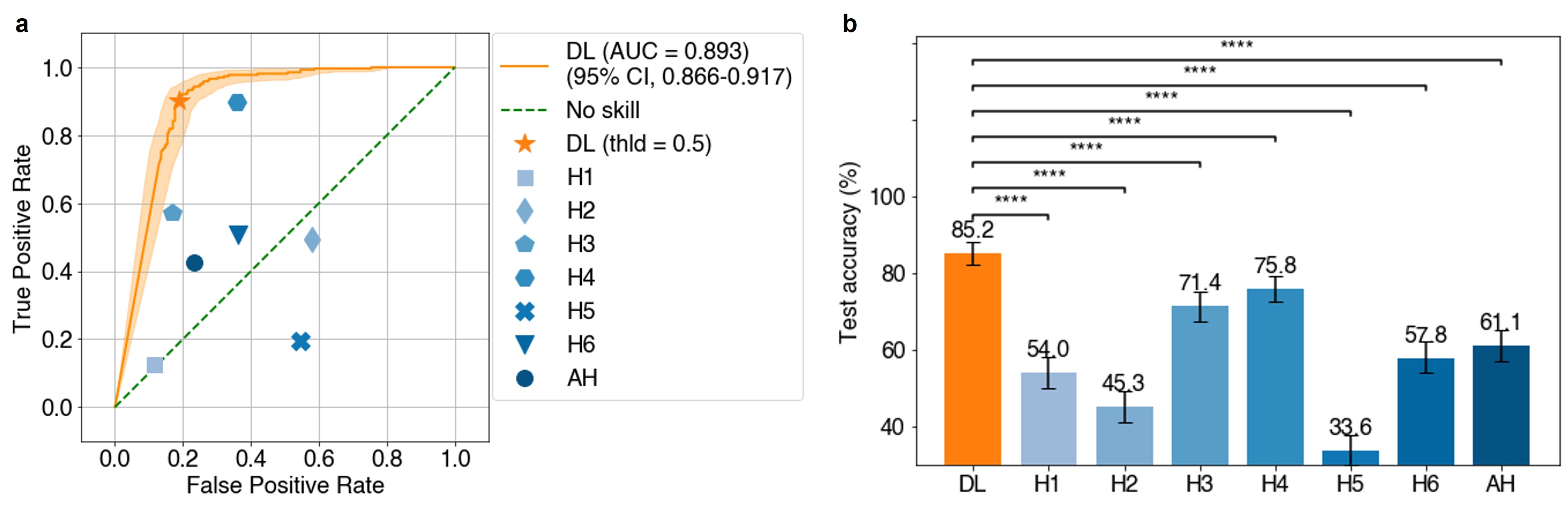


**Figure S3. Results of image classification task by the ensemble deep learning model, six human volunteers, and their average.**

**a** The receiver operating characteristic (ROC) curve of the performance of the ensemble DL model on testing frames. The 95% confidence intervals (CIs) of the ROC curve were indicated as the orange shading area. The orange solid star represents the performance of the ensemble DL model with the default probability threshold (thld) of 0.5 to binarize its output and the blue markers with different shapes and saturation represent the performance of six human volunteers and their average (AH). We applied majority voting to the six predictions on each testing frame to obtain an average human performance. In the case of a tie, we randomly assigned the prediction of before or after onset. **b** Testing accuracy bar chart of the ensemble DL model, six human volunteers and their average (AH), where the error bars represent the 95% CI. The ensemble DL model significantly outperforms each individual human and the average human. **p* < 0.05, ***p* < 0.01, ****p* < 0.001, *****p* < 0.0001, NS, not significant, two-sided *z*-test used in all cases. All the 95% CIs are estimated by bootstrapping the testing dataset with 1000 replicates.


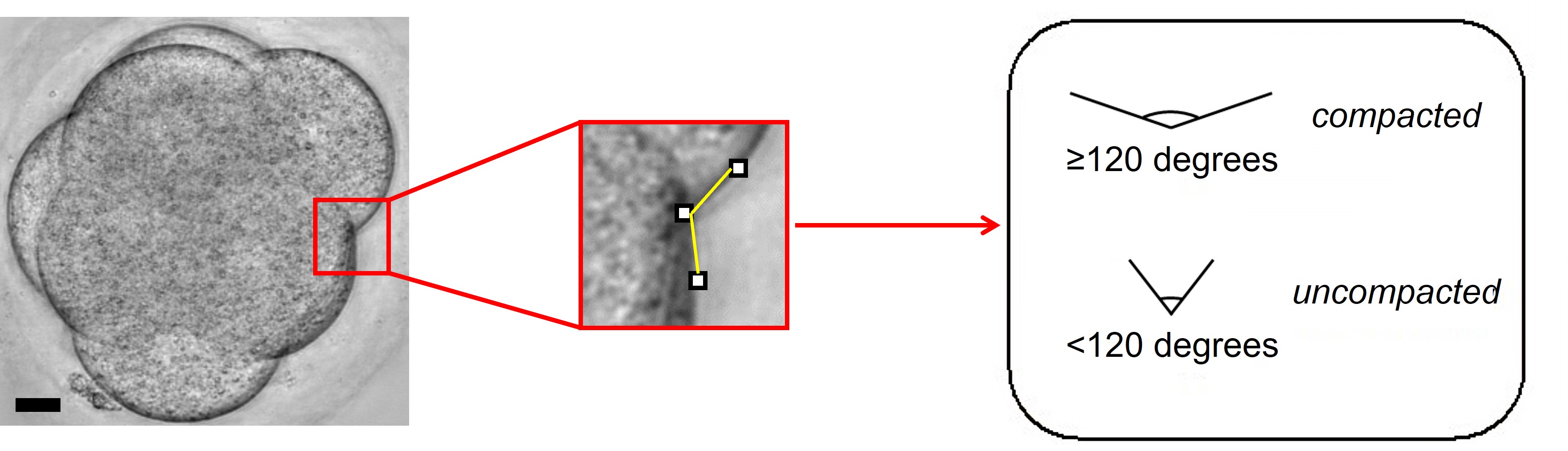


**Figure S4. Criteria for annotating compaction.**

We measured compaction using the inter-blastomere angle. The first time point at which the minimal inter-blastomere angle was ≥ 120 degrees was defined as the compaction point. All frames including and succeeding this point were defined as compacted, whilst all frames prior to this point were defined as uncompacted. Scale bar = 10 *μ*m.


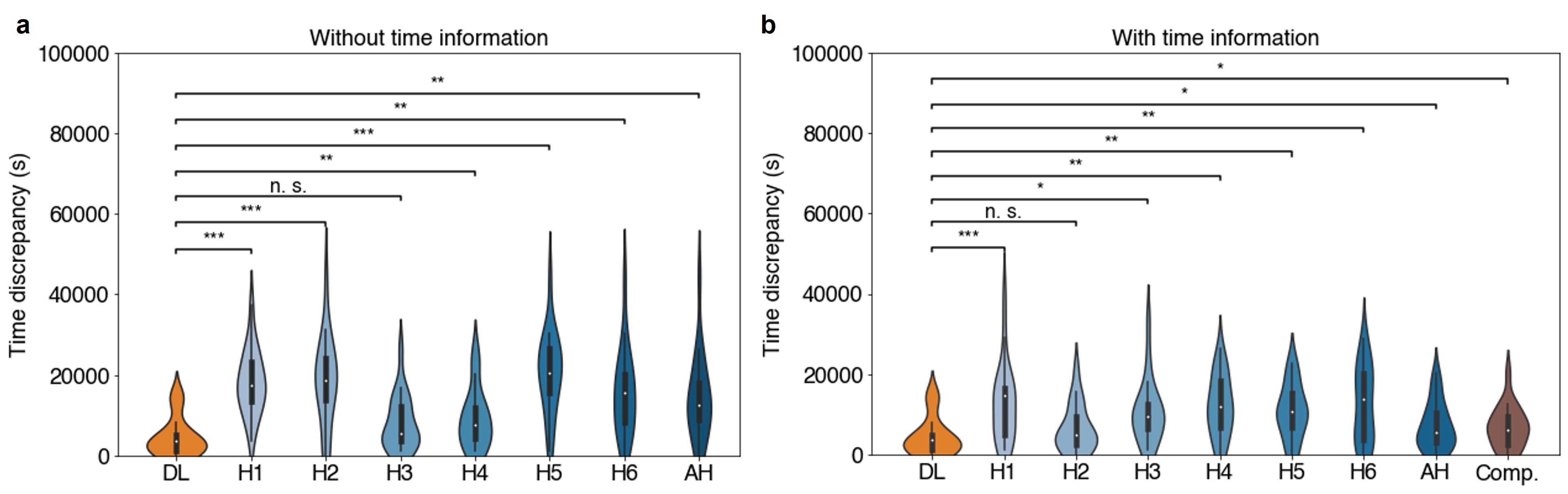


**Figure S5. Comparative analysis of the polarity onset time point prediction by the ensemble deep learning model, six human volunteers, their average, and the compaction proxy.**

The violin plots represent time discrepancies between the annotated and the predicted polarity onset time index of the 19 testing embryos by the ensemble DL model, each of six human volunteers (H1-H6), their average (AH), and the compaction proxy (Comp.). **a** Six human volunteers were given the randomized testing frames without any time information. Their predicted labels were then chronologically ordered for each testing embryo and temporally smoothened to extract the polarity onset time point prediction, as shown in Fig. 2c. Their average result was processed in the same way. **b** Six humans were given the chronologically ordered frames for each testing embryo. They directly estimated the polarity onset time point. Their average result was the arithmetic mean of predicted time indexes for each testing embryo. Comparison between the ensemble DL model and each human is given in the figure. **p* < 0.05, ***p* < 0.01, ****p* < 0.001, *****p* < 0.0001, NS, not significant, two-sided Wilcoxon matched-pairs signed-rank test.


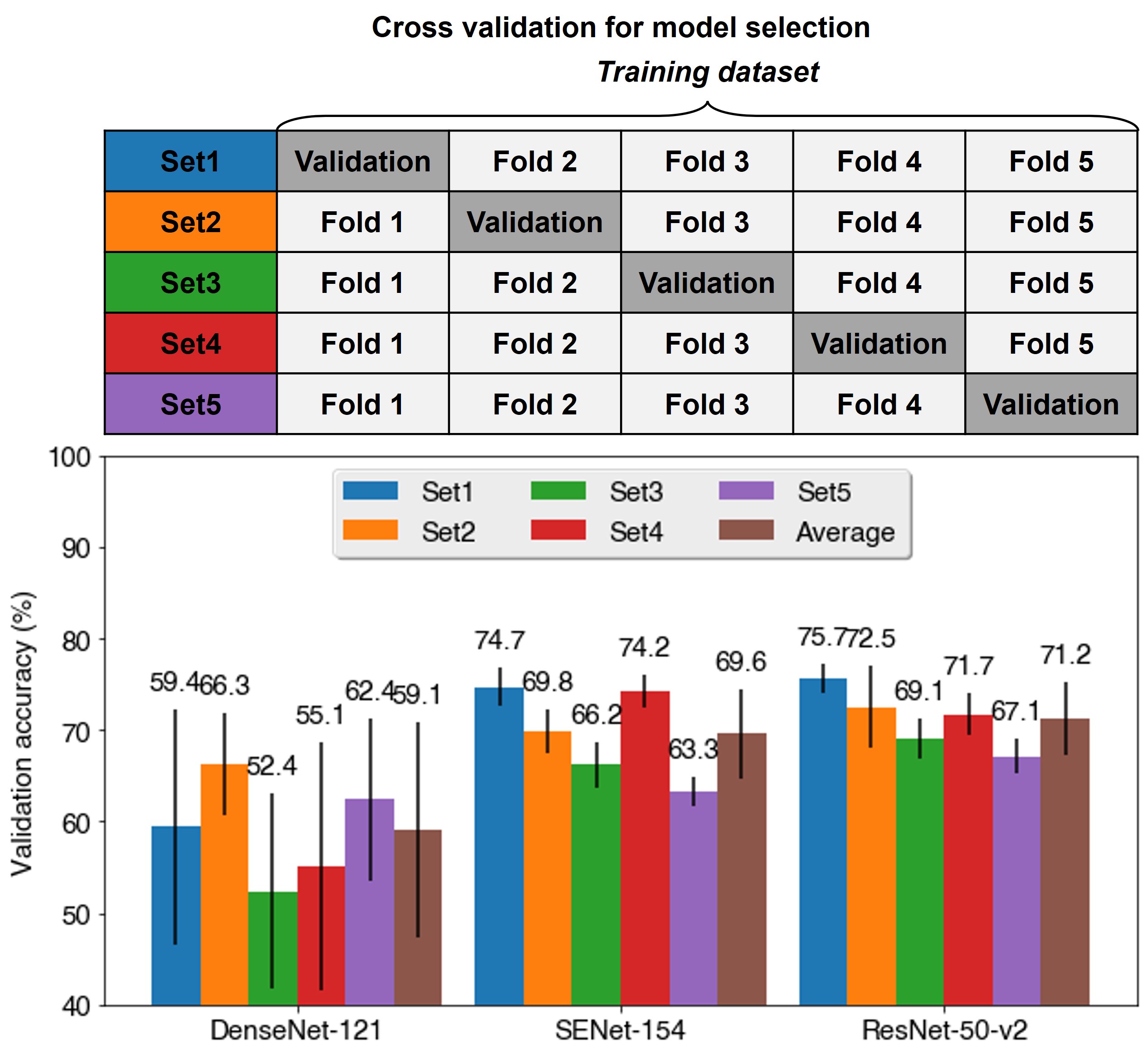


**Figure S6. Comparison among the performance of different image classification DCNN backbones.**

Five-fold cross validation was adopted here to make model selection. The training dataset was evenly split into 5 folds. Then, three backbone models learnt on four folds and were validated on the remaining one. To minimize the variance brought by the optimization setting, we repeated each numerical experiment 5 times. We summarized the validation accuracy of three models on each fold and the total average validation accuracy in the bar chart, where the error bars represent their standard deviation. Both validation accuracy and their standard deviation shows that ResNet-50-v2 is the optimal backbone for our task.


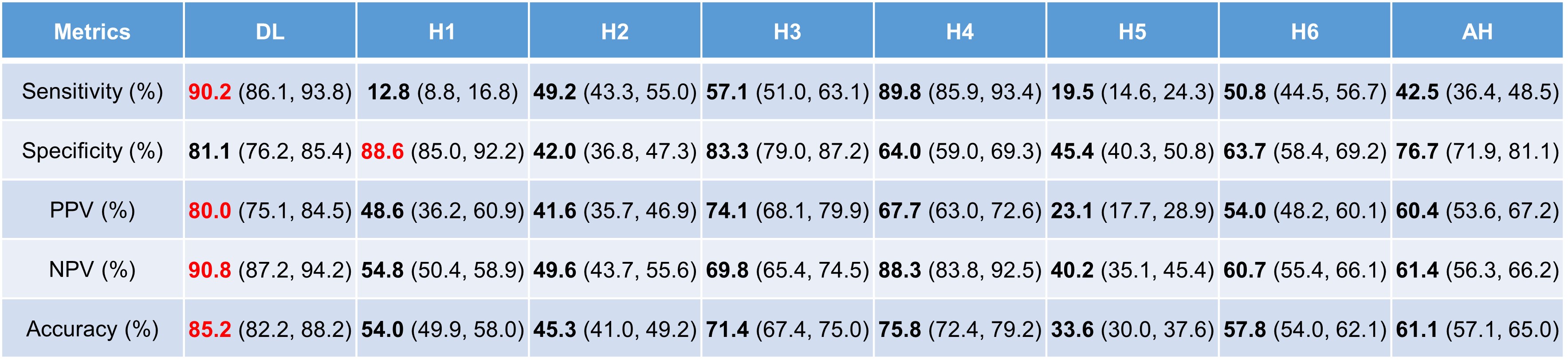


**Table S1. The performance of the ensemble deep learning (DL) model, six human volunteers (H1-H6) and their average (AH) on the testing dataset.**

95% confidence intervals are included in brackets. They are estimated by bootstrapping the testing dataset with 1000 replicates. The highest value of each metric achieved by all predictors is marked in bold red. PPV, positive predictive value; NPV negative predictive value. H1-H3 are females and H4-H6 are males.

**Table S2. Result summary of different ensemble techniques.**

95% confidence intervals are included in square brackets. They are estimated by bootstrapping the testing dataset with 1000 replicates. The highest testing accuracy in each category is marked in bold.

| Varied element | Different techniques | Testing accuracy |
| --- | --- | --- |
| Training data | raw training data | **85.2%** [82.2%, 88.2%] |
|  | 6-fold cross-validation on training data | 83.4% [80.3%, 86.4%] |
|  | bootstrapping training data 6 times | 82.2% [78.7%, 85.4%] |
| Model | varying optimization initials | **85.2%** [82.2%, 88.2%] |
|  | three SENet-154 models + three ResNet-50-v2 models | 83.4% [80.1%, 86.4%] |
| Combination method | averaging the output probability from ensemble members | 85.2% [82.2%, 88.2%] |
|  | applying majority voting on the output labels from ensemble members* | **85.6%** [82.5%, 88.3%] |

* If we meet a tie, the averaged probability will be used to make a judgement instead.
